# P02.120. Qualitative systemic review on Complementary and Alternative Medicine treatments in Inflammatory Bowel Diseases

**DOI:** 10.1186/1472-6882-12-S1-P176

**Published:** 2012-06-12

**Authors:** J Langhorst, R Lauche, H Vorpeil, M Baecker, P Klose, G Dobos

**Affiliations:** 1 University of Duisburg, Complementary and Integrative Medicine, Essen, Germany

## Purpose

We performed a systematic review (with qualitative metaanalysis) for Complementary and Alternative Medicine (CAM) as defined by the National Institute of Health, with the exception of dietary and nutritional supplements, in Inflammatory Bowel Disease (IBD), i.e., Crohn´s disease (CD) and ulcerative colitis (UC).

## Methods

A computerized search of databases (Cochrane Library, Medline, PsychINFO, and Scopus) through June 2011 was performed. We screened the reference sections of original studies and systematic reviews in the English language for CAM in IBD, CD and UC. Randomized controlled trials (RCT) and controlled trials (CT) were included. RCTs comparing treatment to controls were assessed by a methodological quality score.

## Results

A total of 11 RCTs and 4 CTs in herbal therapy (i.e., plantago ovata, boswellia, barley foodstuff, curcumin, tormentil, aloe-vera gel, wheatgrass-juice, evening primrose oil, andrographis paniculata, sophora and wormwood), 1 RCT in trichuris suis ovata, 2 RCTs in mind-body interventions and self-management, 2 RCTs in acupuncture, and 1 RCT in balneotherapy were found. The 17 RCTs had an average study size of 61 patients (range 20 – 126) with a number of groups ranging from two to three. The quality score assessment of the RCTs yielded a mean score of 57 out of 100.

## Conclusion

The average methodological quality of the identified studies was fairly low. Best evidence was found for herbal therapy, i.e. plantago ovata and curcumin in UC maintenance therapy, wormwood in CD, trichuris suis ovata in UC, mind-body therapy and self-intervention in UC, and acupuncture in UC and CD.

